# Daily Lifestyle and Cutaneous Malignancies

**DOI:** 10.3390/ijms22105227

**Published:** 2021-05-14

**Authors:** Yu Sawada, Motonobu Nakamura

**Affiliations:** Department of Dermatology, University of Occupational and Environmental Health 1-1, Iseigaoka, Yahatanishi-ku, Kitakyushu, Fukuoka 807-8555, Japan; motonaka@med.uoeh-u.ac.jp

**Keywords:** daily lifestyle, skin cancer, melanoma, squamous cell carcinoma, basal cell carcinoma, merkel cell carcinoma

## Abstract

Daily lifestyle is a fundamental part of human life and its influence accumulates daily in the human body. We observe that a good daily lifestyle has a beneficial impact on our health; however, the actual effects of individual daily lifestyle factors on human skin diseases, especially skin cancers, have not been summarized. In this review, we focused on the influence of daily lifestyle on the development of skin cancer and described the detailed molecular mechanisms of the development or regulation of cutaneous malignancies. Several daily lifestyle factors, such as circadian rhythm disruption, smoking, alcohol, fatty acids, dietary fiber, obesity, and ultraviolet light, are known to be associated with the risk of cutaneous malignancies, malignant melanoma, squamous cell carcinoma, basal cell carcinoma, and Merkel cell carcinoma. Although the influence of some daily lifestyles on the risk of skin cancers is controversial, this review provides us a better understanding of the relationship between daily lifestyle factors and skin cancers.

## 1. Introduction

Human beings are constantly exposed to various environmental stimuli and have developed their ability to adapt to these external factors, such as circadian rhythm, food intake, ultraviolet light, and microorganisms [1,2,3]. Among these environmental factors on Earth, daily lifestyle is a fundamental part of human life and influences human health. For instance, the influence of lifestyles, such as dietary foods and luxury consumptions, accumulates daily and affects human health [4,5,6]. Therefore, these influences are helpful information for clinicians to understand the detailed molecular pathogenesis of daily lifestyle-related human diseases.

The skin is the outmost layer organ and is one of the most exposed human organs to environmental stimuli [7]. This fact prompts us to speculate that daily lifestyle might well influence the risk of skin diseases. In addition, the skin is composed of various cells, such as keratinocytes, melanocytes, immune cells, and adipocytes [8]. This is the reason why daily lifestyle might broadly influence the risk of cutaneous malignancies.

Recent information on skin cancers gained from novel technologies such as in vivo reflectance confocal microscopy promotes deep understanding of the detailed molecular mechanisms of skin cancer development [9,10,11]. However, its advanced form is intractable and there are still only a limited number of therapeutic options against metastatic lesions of skin tumors. Therefore, how the accumulation of daily lifestyle factors influences skin cancer development is helpful information for clinicians.

In this review, we focused on the daily lifestyle influences on human cutaneous malignancies. First, we introduce representative skin malignancies and then, describe how each factor of daily lifestyle influences the development or regulation of skin malignancies. Ultraviolet light causes DNA damage, which leads to cancer formation and is one of the main risks to skin cancer. The role of ultraviolet light irradiation in the development of skin cancer has already been intensively reviewed in a lot of papers [12]. Therefore, this review only focused on major daily lifestyle factors, including UV irradiation.

### 1.1. Daily Lifestyle-Related Malignancies

Daily lifestyle is closely related to the risk of skin tumors, such as malignant melanoma, basal cell carcinoma, squamous cell carcinoma, and Merkel cell carcinoma. First of all, the characteristics of these malignancies are briefly introduced.

### 1.2. Malignant Melanoma

Melanoma is a malignancy derived from melanocytes, with an unfavorable life-threatening clinical behavior due to its malignant characteristics in addition to a limited number of radical treatments [13,14]. The tumorigenesis of melanoma is associated with several gene mutations in BRAF, NRAS and C-KIT, which are correlated with the histopathological characteristics of melanoma [15,16,17]. One of the most common mutations is the BRAF gene, whose inhibition showed a beneficial antitumor reaction against melanoma [18].

**Figure 1:**
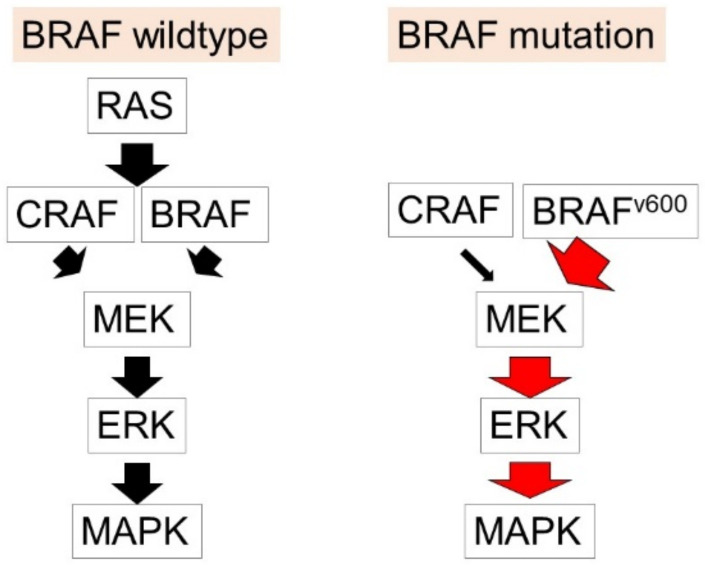
The mechanisms of development of melanoma. In BRAF wild type melanoma, RAS is one of the non-BRAF oncogenes activated in melanoma and promotes cellular functions in melanoma, including the promotion of cell growth and apoptosis. They activate downstream signaling pathways MEK/ERK, MAPK, and the PI3K/AKT pathways. In the BRAF mutation melanoma, BRAF^V600^ mutations activate the downstream pathway of MAPK and exert the development and progression of melanoma.

The BRAF gene is located on chromosome 7 (7q34) and encodes the BRAF protein [19], which is involved in the activation of the mitogen-activated protein kinase (MAPK) pathway [20], leading to the regulation of cell growth, differentiation, proliferation and apoptosis [21]. BRAF has been identified as a commonly mutated gene in human tumors [22]. Mutations in the BRAF gene could cause an impaired protein function [23], and BRAF^V600^ mutations have been detected in nearly 50% of malignant melanoma and activate the downstream pathway of MAPK [24] (Figure 1).

NRAS mutations are the second most frequent mutations in malignant melanoma [25]. RAS proteins control the MAPK and PI3K pathways. C-KIT is also important for the pathogenesis of melanoma, especially acral and mucosal melanoma [26,27]. Cyclin-dependent kinase inhibitor type 2A (CDKN2A) has also been identified as a major gene associated with the risk of melanoma [28]. Although there are severe adverse therapeutic reactions [29,30,31], recent advancements in immunotherapy targeted against PD-1 or CTLA-4 have dramatically improved clinical outcomes in advanced forms of melanoma via inhibition of the escape phenomenon from antitumor immunity, which is mediated by these molecules [32,33,34,35,36]. Although the current development of immune checkpoint inhibitors and BRAF-targeted treatment has improved their clinical outcomes, these therapies do not reach a satisfactory level of clinical outcomes. Circadian rhythm, smoking, alcohol, vitamin A, obesity, fatty acids, coffee/caffeine, and ultraviolet light are known to be associated with the risk of malignant melanoma.

### 1.3. Squamous Cell Carcinoma

**Figure 2:**
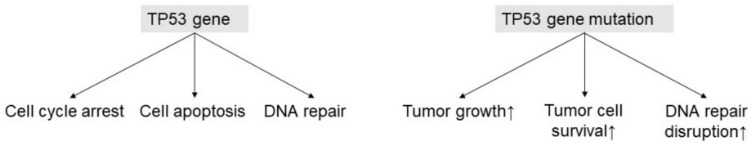
p53 gene and tumor development. A brief schema of p53 and tumorigenesis. p53 gene is responsible for cell cycle arrest, apoptosis, and DNA repair as a tumor suppressor gene. Once p53 gene mutation occurs, these cell functions lose control and develop the tumor by promoting tumor growth, cell survival, and DNA repair disruption.

Cutaneous squamous cell carcinoma is a cutaneous malignancy derived from keratinocytes in the skin and is a highlighted issue for clinicians because of its current increasing incidence rate in the world [37,38]. The advanced metastasis of cutaneous squamous cell carcinoma shows an unfavorable clinical behavior due to there being a limited number of effective treatments [39,40]. A tumor suppressor gene, p53, is closely related with the development of cutaneous squamous cell carcinoma, and mutation of the p53 gene is an important step in the development of SCC [41]. The p53 gene is responsible for cell cycle arrest, apoptosis, and DNA repair as a tumor suppressor gene. Once p53 gene mutation occurs, these cell functions lose control and develop the tumor by promoting tumor growth, cell survival, and DNA repair disruption (Figure 2). Ultraviolet light exposure, circadian rhythm, smoking, alcohol, dietary fiber, citrus intake, obesity, and fatty acids are related to the risk of cutaneous squamous cell carcinoma.

### 1.4. Basal Cell Carcinoma

The nomenclature for basal cell carcinoma is explained by the histomorphological resemblance of cancer cells to basal cells of the epidermis [42]. Although the metastatic form is rare, a local invasion is commonly observed. In addition, mutations in hedgehog pathway genes primarily involve patched homolog (PTCH) and smoothened homolog (SMO); hedgehog pathway inhibition by vismodegib showed an antitumor response against invasive metastatic basal cell carcinoma [43]. Mutations of p53 and the PTCH gene are major candidate tumor suppressor genes for basal cell carcinoma [44]; once UV-induced, these gene mutations are related with the tumorigenesis of basal cell carcinoma [45,46]. In addition, circadian rhythm, smoking, alcohol, furocoumarin intake, obesity, fatty acids, coffee/caffeine, and ultraviolet light are associated with the risk of basal cell carcinoma.

### 1.5. Merkel Cell Carcinoma

Merkel cell carcinoma is an uncommon aggressive cutaneous malignancy with a high rate of local recurrence and distant metastasis, despite radical surgical resection. Recent studies identified that Merkel cell polyoma virus is among the pathogeneses. Polyomavirus-encoded T antigens target several tumor suppressor proteins, such as the retinoblastoma protein (RB) and p53 protein [47]. In particular, large T antigen (LTA) contributes to the tumorigenesis by suppression of the cell cycle regulatory function of RB, while LTA antigens lack a putative p53-binding domain because of tumor-associated LTA mutations [48,49]. On the other hand, there are many UV-mediated mutations in Merkel cell polyoma virus-negative patients [50]. Therefore, among daily lifestyle-related factors, it is thought that UV radiation is one of the major triggers for the development of Merkel cell polyoma virus-negative Merkel cell carcinoma.

## 2. The Daily Lifestyle Associated with the Cutaneous Malignancies

In this section, we introduce the risk of cutaneous malignancies associated with representative daily lifestyles, such as sleeping, smoking, alcohol intake, dietary fiber, obesity, fatty acid, coffee/caffeine, vegetables, and ultraviolet light. In addition, the detailed molecular mechanisms of advancement or regulation of cutaneous malignancies in some parts of daily lifestyle factors are also described.

### 2.1. Circadian Rhythm

A day on earth is around 24 h; however, the internal clock period of the human body is closer to 25 h [51]. Therefore, adaptation to the earth’s daily cycle is essential for animals living on Earth, and the human body has been developed to adjust to the Earth’s daily cycle period through regulation of the circadian rhythm. Disruption of the circadian rhythm negatively affects human health, which is becoming a global health threat, including metabolic and immune diseases [52]. Since the circadian rhythm regulates daily fluctuations in immune response, it is assumed that circadian disruption results in an impaired tumor-associated immune response and facilitates tumor growth. In fact, the epidemiological study identified that night shift work is associated with a high risk of several cancers [53]. Shift work with circadian disruption is also associated with the risk factor for cutaneous malignancies.

#### 2.1.1. Melanoma and Circadian Rhythm

Recent systematic review analysis revealed that shift work is associated with an increased risk of melanoma [54]. As detailed molecular mechanisms, circadian clock gene expression depends on the development of melanoma. Disruption of the circadian rhythm developed tumor growth in a murine melanoma model and impaired inflammatory reaction in M1 macrophages-mediated antitumor immunity in a mouse experiment [55]. Interestingly, circadian regulation genes are reduced in melanoma [56,57,58]. The expression of the clock gene aryl hydrocarbon receptor nuclear translocator-like protein 1 (BMAL1) shows a positive correlation with the overall survival, T-cell activity, and the beneficial impact of immune checkpoint inhibitors [59]. Although the clock genes are suppressed in melanoma, treatments of circadian rhythmicity by dexamethasone trigger the recovery of rhythmic clock and cell cycle gene expression, which result in low frequency in the S phase tumor cell and high frequency in the G1 phase. In addition, silencing BMAL1 impaired the effects of dexamethasone on tumor growth of melanoma in an in vitro experiment [60]. Clock gene disruption in melanoma is associated with decreased light-dependent activation of DNA repair genes [61].

#### 2.1.2. Squamous Cell Carcinoma/Basal Cell Carcinoma and Circadian Rhythm

A recent systematic review analysis of the relationship between skin cancer and shift work revealed that shift work is associated with a significant decreased risk of basal cell carcinoma [54]. However, this systematic review could not detect a significant difference between shift work and the risk of squamous cell carcinoma. Although there are a limited number of studies focused on the role of clock genes in cutaneous squamous cell carcinoma, squamous cell carcinoma originating from the oral and head/neck, not from the skin, show an increased risk of tumor development by disruption of the circadian rhythm. Abnormal expression of the clock gene PER1 shows a correlation with the tumorigenesis of squamous cell carcinoma [62]. PER1 is decreased in squamous cell carcinoma [63,64] and low expression of PER1 is closely related to unfavorable clinical behavior, such as developed lymph node metastasis and advanced clinical stage [64,65], and poor survival [66]. Consistently silencing PER1 in melanoma cells promotes tumor proliferation [62,67,68] and development in an in vitro experiment [69], while overexpression of PER1 enhances the apoptosis of squamous cell carcinoma [64]. PER2 expression is also decreased in squamous cell carcinoma [70,71] and decreased PER2 expression is associated with advanced clinical stage, lymph node metastasis, and unfavorable patient survival [70]. Silencing PER2 in melanoma cells reduces the apoptosis of tumor cells [72,73,74] and induces tumor development in an in vitro experiment [69]. Consistently high expression of PER2 negatively regulates tumor development [71]. The expression of PER3 is significantly downregulated in the tumor [66], which is associated with advanced tumor stages, tumor size, tumor invasion, and unfavorable patient survival [66]. The circadian clock gene BMAL1 suppresses tumor development in tongue squamous cell carcinoma [66,75]. The degree of BMAL1 expression is related to the clinical course and BMAL1 expression is downregulated in tongue squamous cell carcinoma, leading to a high frequency of tumor cell invasion and metastasis [75]. Consistently, the suppression of BMAL1 results in the development of the tumor [69].

### 2.2. Smoking

Tobacco smoking is a representative daily lifestyle-related habit, which has a long history in human beings [76]. Although a large population still continues this habit in their daily lifestyle, tobacco smoking is also recognized worldwide as a harmful substance for human health that causes various organ disorders and malignancies [77,78,79]. The skin is also known to be involved in smoking-related human diseases.

#### 2.2.1. Melanoma and Smoking

Although smoking is negatively associated with melanoma incidence [80,81,82], smoking is not associated with melanoma-specific mortality [82] or sentinel lymph node metastasis [83]. On the contrary, another study also identified that current smoking is associated with sentinel lymph node metastasis and tumor ulceration [84]. Never smoking is associated with decreased tumor thickness in melanoma [84].

#### 2.2.2. Squamous Cell Carcinoma/Basal Cell Carcinoma and Smoking

The influence of smoking on the risk of squamous cell carcinoma is controversial. Some studies showed that current smoking is associated with an increased incidence of squamous cell carcinoma [85,86,87], while other studies could not find a significant influence of smoking on the risk of squamous cell carcinoma in smokers [88,89].

The influence of smoking on the risk of basal cell carcinoma is also controversial. Current smoking and heavy smoking conditions are associated with a decreased risk of basal cell carcinoma [90,91,92,93], while several studies could not detect a significant risk of basal cell carcinoma in smokers [85,86,94,95,96,97,98]. However, the prevalence of clinical subtype of basal cell carcinoma might be associated with smoking. One study identified that morphea-type basal cell carcinoma exhibits a significantly high frequency in smokers compared to solid basal cell carcinoma [99].

### 2.3. Alcohol

Alcohol is one of the major human daily lifestyle-related factors with a long-lasting history of being a habit in humans. Since alcohol intake is a common lifestyle choice in the world, it is important to understand the actual effect of alcohol on human diseases in daily clinical practice. Indeed, there are many alcohol-related influences on human diseases that have both beneficial and detrimental effects. Cutaneous malignancies are also influenced by alcohol intake.

#### 2.3.1. Melanoma and Alcohol

The risk of alcohol in melanoma was controversial in each individual statistical analysis [100,101,102,103,104,105,106,107,108]; however, a meta-analysis that included 16 studies with a total of 6251 cases of cutaneous melanoma revealed that the relative risk of malignant melanoma is increased in the alcohol intake group [109].

#### 2.3.2. Squamous Cell Carcinoma/Basal Cell Carcinoma and Alcohol

The risk of basal cell carcinoma in alcohol intake was controversial in each individual statistical analysis [96,110,111,112,113]. However, a systematic review identified evidence that alcohol drinking is closely related to the risk of basal cell carcinoma in a dose-dependent manner [114]. The systematic literature studies were case–control or cohort studies that examined alcohol intake and risk of BCC and a total of 91,942 basal cell carcinoma patients and 3299 cutaneous squamous cell carcinoma patients were investigated. Alcohol intake showed a positive correlation with the risk of basal cell carcinoma and squamous cell carcinoma.

### 2.4. Dietary Fiber/Vegetables/Fruits

Dietary fiber is a non-digestible substance consistent with the plant cell wall. Dietary fiber includes important plant materials for human health, such as polysaccharides, oligosaccharides, and lignin [115]. We can commonly intake dietary fiber by eating vegetables; however, the consumption of a fiber-rich diet is currently decreasing. Current studies showed dietary fiber substances, vegetables and fruits have beneficial effects on human health. Therefore, these influences are important for clinicians to better understand the regulation of cutaneous malignancies.

#### 2.4.1. Melanoma and Dietary Fiber/Vegetables/Fruits

Several studies identified a significant negative correlation between vitamin A intake and melanoma risk [108,116,117]. However, no significant association is observed between furocoumarins consumption and the risks of melanoma [118]. Dietary fiber-derived short-chain fatty acids are produced from bacteria under anaerobic conditions and have various bioactive actions against immune cells and epithelial cells [2]. Dietary fiber-derived short-chain fatty acid butyrate promotes melanoma cell invasion by the induction of Annexin A1 (ANXA1), which negatively regulated E-cadherin expression in an in vitro experiment [119].

#### 2.4.2. Squamous Cell Carcinoma/Basal Cell Carcinoma and Dietary Fiber/Vegetables/Fruits

A higher intake of total furocoumarins is associated with a high risk of basal cell carcinoma [118,120,121]. There is no association between citrus fruits intake and the risk of cutaneous squamous cell carcinoma [118,121,122]. One study identified a negative correlation between a high total citrus intake and the risk of squamous cell carcinoma [120]. An epidemiological study showed that fiber intake is negatively associated with the risk of esophagus squamous cell carcinoma [123].

### 2.5. Obesity

Since human beings have developed into a modern civilization, we can easily obtain enough food and do not necessarily require physical activity due to the development of transportation. An excessive food intake or insufficient physical activity causes obesity, which develops various physiological and pathological changes in the human body due to disruption of the energy balance. Therefore, obesity-related human diseases are highlighted for clinicians. Current studies identified that obesity is closely related to the development of cutaneous malignancies.

#### 2.5.1. Melanoma and Obesity

The relationship between obesity and the risk of melanoma is controversial. One study reported that obesity is significantly associated with a risk of melanoma thickness [124]; however, several studies could not find a relationship between the risk of melanoma and obesity [125,126,127]. Obesity significantly increases the risk of melanoma in individuals younger than 50 years old, whereas this association is not significant in people over 50 years old [128]. Body mass index at age 20 is significantly associated with the risk of melanoma [129].

#### 2.5.2. Squamous Cell Carcinoma/Basal Cell Carcinoma and Obesity

Body mass index in the obese range showed a 32% lower risk of squamous cell carcinoma compared to that in the normal range [127]. Another study showed a high relative risk of squamous cell carcinoma in obese females only [126]. Obese female patients showed a decreased risk of basal cell carcinoma [126]. In another study, a body mass index in the obese range showed a 19% lower risk of basal cell carcinoma [127].

### 2.6. Fatty Acids

A fatty acid is a carboxylic acid with a long aliphatic chain, which is either a saturated or unsaturated fatty acid [6]. Fatty acids are important dietary sources of fuel for animals and are essential structural components for cells. Recent studies identified that fatty acids have bioactive actions in the human body with physiological and pathological effects. In the skin, fatty acids, such as prostaglandins and leukotrienes, positively and negatively drive inflammatory skin diseases and contribute to the development of cutaneous malignancies [130,131,132,133]. Furthermore, omega-3 fatty acids and their metabolites derived from fish and nuts have a beneficial impact on human skin diseases [134,135,136].

#### 2.6.1. Melanoma and Fatty Acids

The “high-fish, low-meat, and low-fat” dietary pattern is not associated with the risk of melanoma thickness [133]. The risk of melanoma increases with the intake of omega-6 fatty acids [137] and decreases with the intake of omega-3 fatty acids [138]. Tumor growth is suppressed by omega-3 fatty acids [139], such as DHA [140,141]. Tumor invasion in melanoma is promoted by arachidonic acid [142] and PGE2 [142], while this is suppressed by omega-3 fatty acids [143], such as EPA [142] and DHA [142].

Several experimental studies identified the detailed molecular relationship between tumor regulation and fatty acids. DHA reduces the migration/invasion of melanoma by down-regulating several matrix metalloproteinases, such as MMP-2 and MMP-13, which are involved in melanoma invasion [141]. DHA and EPA enhance cisplatin-induced inhibition of tumor growth and migration [144]. Omega-6 fatty acids induce CXCR4 expression in melanoma, although ω3 fatty acids decrease CXCR4 expression, leading to the prevention of melanoma metastasis [145]. Leukotriene B4 (LTB4), which induces growth of melanoma cells, and an LTB4 receptor antagonist inhibit acute inflammation-associated tumor growth [146]. The eicosapentaenoic acid-derived leukotriene, leukotriene B5, significantly suppresses the development of tumors [146]. DHA metabolites Resolvin D1 (RvD1) and Resolvin D2 (RvD2) suppress melanoma tumor cell growth [147], and the metastasis of melanoma cells.

#### 2.6.2. Squamous Cell Carcinoma/Basal Cell Carcinoma and Fatty Acids

A high intake of omega-3 PUFA shows a substantially reduced risk of squamous cell carcinoma [148], and a high plasma level of EPA concentration is associated with a lower risk of squamous cell carcinoma [149]. Arachidonic acid intake has a marginally increased risk of squamous cell carcinoma [149]. On the contrary, those with high omega-6 fatty acid and α-linolenic acid intakes show a significantly lower risk of basal cell carcinoma [148,149]. A decreased risk of basal cell carcinoma is associated with a higher serum concentration of total omega-6, linoleic acid, and linolenic acid serum concentrations [150]. A higher omega-6 fatty acids intake is associated with the risk of basal cell carcinoma [137]; however, the association between omega-3 fat intake and the risk of squamous cell carcinoma and basal cell carcinoma could not be detected by a meta-analysis [138].

Several experimental studies showed the beneficial potential of omega-3 fatty acids on the regulation of squamous cell carcinoma. Omega-3 fatty acids exhibit the inhibition of tumor growth of both basal cell carcinoma and squamous cell carcinoma and modulate the immune response [151]. A DHA metabolite RvD2 has antitumor effects against squamous cell carcinoma [152]. RvD2 shows anti-inflammatory action and suppresses inflammatory cytokines and chemokines by tumor cells.

### 2.7. Coffee/Caffeine

Drinking coffee is a broadly familiar human daily lifestyle factor and habit in the world. Coffee consists of more than 1000 components, responsible for its aroma and flavor, and shows physiological and pathological activities to the human body [153]. Coffee and caffeine are closely related to the development of cutaneous malignant tumors.

#### 2.7.1. Malignant Melanoma and Coffee/Caffeine

Coffee and caffeine are associated with a low risk of malignant melanoma [154,155,156], which is more apparent for melanomas occurring on body sites with higher continuous sun exposure (head, neck, and extremities) [156]. Consistently, the meta-analysis also supports the finding of a low risk of melanoma through coffee consumption [157], but not decaffeinated coffee [158]. One study reported that coffee consumption is negatively associated with melanoma risk among men, but not among women [159].

#### 2.7.2. Squamous Cell Carcinoma/Basal Cell Carcinoma and Coffee/Caffeine

Caffeine intake decreases the risk of basal cell carcinoma [160,161]. Combined caffeinated coffee plus hot tea consumption is also negatively associated with the risk of basal cell carcinoma [162], while there is no association between decaffeinated coffee consumption and the risk of basal cell carcinoma [163]. However, one study could not detect the association between total caffeine intake and incidence of basal cell carcinoma or squamous cell carcinoma [160].

### 2.8. Ultraviolet Light Exposure

The skin is necessarily exposed to sunlight and a part of it is ultraviolet light, which is classified as UVA, UVB and UVC. UVB damages DNA more effectively than UVA. Ultraviolet light is responsible for damage to DNA and gene mutations, including mutations of the p53 gene [164].

#### 2.8.1. Melanoma and Ultraviolet Light

Ultraviolet light exposure is a risk factor of melanomagenesis [165,166]. Ultraviolet light-induced DNA damage is mediated by p53, whose functional deletion drives ultraviolet light-mediated melanoma development [167]. p53 was also shown to cooperate with BRAF^V600^ mutation to induce melanoma in the presence of ultraviolet light [168]. Interestingly, among BRAF^600^ mutations, the BRAF^600k^ mutation is significantly correlated with ultraviolet light exposure compared with the BRAF^600E^ mutation [169].

#### 2.8.2. Squamous Cell Carcinoma and Ultraviolet Light

Ultraviolet light radiation is one of the triggers of the development of cutaneous squamous cell carcinoma [170] and increases risk of it [45]. The mechanism is thought to be UVB-induced inactivation of p53, accounting for approximately 58% of cutaneous squamous cell carcinoma [171]. In fact, p53-deficient mice showed the development of cutaneous squamous cell carcinoma due to UVB exposure [172].

#### 2.8.3. BCC and Ultraviolet Light

Ultraviolet light increases the risk of basal cell carcinoma [45]. Ultraviolet light causes p53 mutation, which is associated with an increased risk of basal cell carcinoma [46]. Mutations in hedgehog pathway genes primarily involve the genes encoding PTCH and SMO. UVB radiation increases the gene expression of Ptch2, Smo and Gli1 [173].

#### 2.8.4. Merkel Cell Carcinoma and Ultraviolet Light

The gene expression of LTA increases after UV radiation [174] and ultraviolet light radiation increases the risk of Merkel cell carcinoma [165]. There is a greatly increased incidence of Merkel cell carcinoma among fair-skinned individuals compared to its incidence in those with darker skin [50]. The tumor develops on sun exposure site skin such as the face, scalp, and arms [175].

## 3. Recommendations

Although there are controversial results in some parts, we summarized the risk of skin cancer by daily lifestyle factors (Table 1). Circadian rhythm disruption increases the risk of melanoma and decreases the risk of basal cell carcinoma. Smoking increases the risk of squamous cell carcinoma, but decreases the risk of melanoma and basal cell carcinoma. Obesity decreases the risk of basal cell carcinoma, but increases the risk of melanoma. Omega-3 fatty acids decrease the risk of melanoma, squamous cell carcinoma, and basal cell carcinoma, and omega-6 fatty acids increase the risk of melanoma and squamous cell carcinoma. Coffee/caffeine decreases the risk of melanoma and basal cell carcinoma. Ultraviolet light exposure increases the risk of melanoma, squamous cell carcinoma, basal cell carcinoma, and Merkel cell carcinoma.

## 4. Conclusions

This review presents the influence of daily lifestyle on skin malignancies. Daily lifestyle has diverse aspects and there is a possibility that daily lifestyle factors act in an integrated complex manner. In addition to the physiological and pathological effects of a single daily lifestyle factor, knowledge of the more multifaceted effects of daily lifestyle might be necessary to obtain a better understanding of actual daily lifestyle influence on cutaneous malignancies. Furthermore, there are largely unknown molecular mechanisms in cutaneous malignancies, depending on daily lifestyle. Therefore, other daily lifestyle factors might also be involved in the pathogenesis of cutaneous malignancies. Daily lifestyles are changing overtime. Further investigation of the detailed mechanisms of the development of cutaneous malignancies is desired in order to provide more clarity.

## Figures and Tables

**Table 1:** The relationship between daily lifestyle factors and the risk of skin cancers.

	Melanoma	Squamous Cell Carcinoma	Basal Cell Carcinoma	Merkel Cell Carcinoma
Circadian rhythm disruption	Risk up [54]	No association [54]	Risk down [54]	
Smoking	Risk down [80,81,82]	Risk up [85,86,87] No association [88,89].	Risk down [90,91,92,93] No association [85,86,94,95,96,97,98].	
Alcohol	Risk up [109]	Risk up [114]	Risk up [114]	
Dietary fiber/vegetables/fruits	Vitamin A: Risk down [108,116,117] Furocoumarins: No association [118]	Citrus: Risk down [120] No association [118,121,122]	Furocoumarins: Risk up [118,120,121]	
Obesity	Risk up [124,128,129] No association [125,126,127]	Risk up [126] Risk down [127]	Risk down [126,127]	
Fatty acids	omega-3 fatty acid: Risk down [138] omega-6 fatty acid: Risk up [137]	Omega-3 fatty acid: No association [138] Omega-6 fatty acid: Risk up [149]	Omega-3 fatty acid: No association [138] Omega-6 fatty acid: Risk up [137]	
Coffee/caffeine	Risk down [154,155,156]		Risk down [160,161,162]	
Ultraviolet light	Risk up [165,166]	Risk up [45,170]	Risk up [45,46]	Risk up [165,176]
